# *Cryptosporidium* Infections in Neonatal Calves on a Dairy Farm

**DOI:** 10.3390/microorganisms12071416

**Published:** 2024-07-12

**Authors:** Michaela Kaduková, Andrea Schreiberová, Pavol Mudroň, Csilla Tóthová, Pavel Gomulec, Gabriela Štrkolcová

**Affiliations:** 1 Department of Epizootiology, Parasitology and Protection of One Health, University of Veterinary Medicine and Pharmacy in Košice, Komenského 73, 040 01 Košice, Slovakia; michaela.kadukova@student.uvlf.sk (M.K.); gabriela.strkolcova@uvlf.sk (G.Š.); 2 Clinic of Ruminants, University of Veterinary Medicine and Pharmacy in Košice, Komenského 73, 040 01 Košice, Slovakia; pavol.mudron@uvlf.sk (P.M.); csilla.tothova@uvlf.sk (C.T.); pavel.gomulec@student.uvlf.sk (P.G.)

**Keywords:** calves, cryptosporidium, ELISA, nested PCR, Ziehl–Neelsen staining method

## Abstract

This study was conducted with the aim of the molecular identification of the protozoan parasite *Cryptosporidium* spp. in calves in the early stage of their development on a dairy farm in Eastern Slovakia. Twenty-five Holstein and Holstein cross calves were included in the study and monitored from their birth to the fifth week of life (1–5 weeks). Fresh fecal samples were collected from the same group of calves each week, except during the fourth week, and with the exception of Sample 8. All samples were analyzed using the Ziehl–Neelsen staining method and coproantigen was tested using the ELISA test as the screening method. Using the ELISA method, the highest incidence of cryptosporidiosis was observed in the second week of life of the calves, while the antigen was detected in 21 (91.6%) calves. Using the Ziehl–Neelsen staining method, the highest incidence was also observed in the second week, with an incidence rate of 62.5%. Positive isolates confirmed by the ELISA test were molecularly characterized. The species and subtypes of *Cryptosporidium* in the positive isolates were identified using PCR and the sequence analysis of the small subunit of the ribosomal 18S RNA (ssu rRNA) and the 60 kDa glycoprotein (gp60) genes of the parasite. The sequence analysis of 29 isolates at the 18S rRNA loci confirmed the presence of two species—*Cryptosporidium parvum* and *Cryptosporidium ryanae*. Out of 29 isolates, 25 were assigned to the species *C. parvum*, with the gp60 locus identified as genotype IIaA17G1R1. Among the individual animal groups, calves are the most common reservoirs of the *C. parvum* zoonotic species. This disease has significant public health implications as contact with livestock and their feces and working with barn manure are major sources of infection, not only for other animals but also for humans.

## 1. Introduction

*Cryptosporidium* is a coccidial parasite currently classified in the Alveolata phylum, *Eimeria* suborder, Cryptosporidiidae family. It attacks the respiratory and digestive systems of reptiles, birds, and mammals. Cryptosporidiosis is a zoonotic disease that causes significant economic losses in the cattle population, especially in calves and young animals [1,2]. To date, more than 51 species of *Cryptosporidium* with 120 genotypes have been recognized, with 19 species and 4 genotypes reported in humans, including *Cryptosporidium hominis*, *Cryptosporidium parvum*, *Cryptosporidium meleagridis*, *Cryptosporidium canis*, and *Cryptosporidium felis* as the most common ones [3,4]. The first case of *Cryptosporidium* in calves was reported in 1971 in Oklahoma in an 8-month-old calf with diarrhea, weakness, and a chronic course. The histopathological findings corresponded to villous atrophy with the presence of various developmental stages of *Cryptosporidium* in the epithelium of the small intestine [5]. In cattle, the infection is most commonly caused by species like *C. parvum*, *C. bovis*, *C. ryanae*, and *C. andersoni* [6]. *C. parvum* usually occurs in pre-weaning calves, while *C. bovis* and *C. ryanae* species are mostly diagnosed in post-weaning calves and young cattle. *C. andersoni* is the most common species infecting adult cattle [7,8,9].

Several studies indicate that diarrhea caused by *Cryptosporidium* species is the main cause of calf mortality in the first three weeks of their life. It is characterized by watery, profuse diarrhea, which may be associated with dehydration, anorexia, poor growth development, and low weight gain, with a high mortality rate in untreated calves, leading to significant economic losses [1,10,11]. Infections in older animals are usually subclinical but can still have a negative impact on production, with lower body condition scores, lower carcass weights, and poor carcass-processing hygiene [12]. Enterotoxigenic *Escherichia coli* K99/F5, rotavirus, and coronavirus, along with *Cryptosporidium*, are the four most important enteropathogens causing neonatal diarrhea in calves worldwide [13,14,15]. The infection primarily spreads through the fecal–oral route, which is considered the main route of transmission, or indirectly through the consumption of contaminated food or water with infectious oocysts [3,16]. The high shedding intensity of environmentally resistant oocysts leads to strong environmental contamination and increases the risk of infection. Infected calves can excrete up to 6 × 10^6^ oocysts per gram of feces, but even a small number of oocysts is sufficient to cause an infection [17]. It is reported that oocyst shedding begins 4 days after birth and peaks on days 7–18, while its intensity decreases after approximately 3 weeks [18].

The predominant species responsible for up to 70% of human cryptosporidiosis cases is *C. hominis*, while *C. parvum* is considered the most common cause of zoonotic infections in humans. Within the *C. parvum* species, more than 20 subtypes with geographical variations have been identified, with subtypes IIa, IIc, and IId showing host adaptation. Subtype IIc appears to be exclusively anthroponotically transmitted, while subtypes IIa and IIb are zoonotic and are mostly found in cattle, sheep, and goats [3,19].

Cryptosporidiosis is challenging to control in both animals and humans due to the environmentally stable oocysts, low infectious doses, and wide range of susceptible hosts. Among the available methods for the diagnosis of *Cryptosporidium*, staining methods, such as Kinyoun or Ziehl–Neelsen staining, as well as immunological methods in the form of commercially available ELISA tests, are well known. Molecular methods based on PCR and DNA sequencing, PCR-RFLP, qPCR, or multiplex PCR are more sensitive than microscopy and immunological detection methods [6,20]. 

Currently, there are neither vaccines nor effective chemotherapeutics known for the control of bovine cryptosporidiosis [9,21]. From an economic and health perspective, it is important to increase the awareness among individuals, especially those who work with ruminants, considering that these animals are often reservoirs of zoonotic species. 

A case report from Slovakia in 2020 confirmed a case of cryptosporidiosis caused by the species *C. parvum*, subtype IIdA15G1, in a veterinary medicine student who worked on a calf farm in the eastern part of Slovakia and was aware of their inadequate hand hygiene after handling calves [22]. This study presents the 5-week monitoring of *Cryptosporidium* oocyst shedding in calves from birth and demonstrates that the second week of a calf’s life is the most critical in terms of the risk of infection. It also focuses on the molecular identification and genotyping of *Cryptosporidium* spp. in calves in the early stage of their development on a dairy farm in Eastern Slovakia. The confirmed genotype IIaA17G1R1 has been identified in Holland and Slovenia in humans, indicating that cattle are a potential source and important reservoir of *C. parvum* infection for both humans and animals in Europe [23,24]. 

## 2. Materials and Methods

This research was conducted from September 2022 to October 2022 on a dairy farm breeding dairy cows, located in Eastern Slovakia. There were no other animals on the farm besides cows, which were under veterinary supervision, vaccination schedules against Infectious Bovine Rhinotracheitis (IBR) were followed. The study included 25 neonatal dairy calves (Holstein and Holstein cross). The calves’ mothers were impregnated through artificial insemination, and the number of calves included in the study was determined based on the expectation that they would calve in the same week. The calving of the mothers took place in birthing pens, and the calves were separated from their mothers immediately after birth into individual pens, which were regularly cleaned and disinfected. The newborn calves were fed with a commercial milk replacer and then with commercial concentrates, with free access to water. Fresh fecal samples were collected from the same group of calves each week from the ground after defecation, from their birth until the fifth week of life (1st week (0–7 days old), 2nd week (8–14 days old), 3rd week (15–21 days old), 4th week (22–28 days old), 5th week (29–35 days old)), except for the fourth week, when sampling was not possible. During the analyzed period, 24 calves were tested, except for the 1st week, in which 25 calves were examined. Sample 8 was tested in the 1st week only because the calf was then relocated to an isolated area due to another non-infectious disease; therefore, sampling was impossible. Samples were collected into clean plastic containers, labeled with the calf’s identification number and date of birth and the date of collection, and transported in a polystyrene thermobox designed for the transportation biological material to the Department of Epizootiology, Parasitology, and Protection of One Health at the University of Veterinary Medicine and Pharmacy in Košice.

Fecal samples were analyzed using a flotation method with a flotation solution of specific gravity 1.3 g/cm^3^ to detect the oocysts of protozoan parasites and the eggs of helminths. Additionally, a zinc sulfate solution with specific gravity of 1.18 g/cm^3^ was utilized to detect cysts of *Giardia duodenalis* [25,26]. Oocysts of *Cryptosporidium* spp. were diagnosed by performing fecal smears; three smears were performed from each fecal sample, stained using the Ziehl–Neelsen staining method, and examined under a microscope after drying [25,27]. One of the examination methods also included the detection of coproantigen using a commercially available kit, CRYPTOSPORIDIUM (FAECAL), Diagnostic Automation, INC, Calabasas, USA. Specific antigens of *Cryptosporidium* spp. in animal feces were detected according to the manufacturer’s instructions. For the ELISA test, positivity was detected above an OD value of 0.15, while all samples below OD 0.149 were considered negative. Specific antigens of *Cryptosporidium* spp. in animal feces were detected according to the manufacturer’s instructions. Fecal samples from calves were examined using both methods in each of these weeks; subsequently, positive samples confirmed by the ELISA method were molecularly identified using the nested PCR method.

### Molecular Analysis of Cryptosporidium Species 

Positive samples detected by the ELISA method were used for DNA extraction using the ZR Fecal DNA MiniPrep Kit (Zymo Research, Tustin, CA, USA) following the manufacturer’s instructions. The genomic DNA was stored at −20 °C and later used for PCR analysis. 

We performed the molecular species identification of *Cryptosporidium* using the nested PCR amplification of the small ribosomal subunit rRNA gene (18S rRNA). In the first step, we used a set of primers, forward 18-F1 (5′-TTCTAGAGCTAATACATGCG-3′) and reverse 18-R1 (5′-CCCTAATCCTTCGAAACAGGA-3′), resulting in a primary product of 1350 bp. The amplification program included initial denaturation at 94 °C for 3 min, followed by 35 cycles of denaturation at 94 °C for 45 s, annealing at 55 °C for 45 s, extension at 72 °C for 60 s, and final extension at 72 °C for 7 min. In the second reaction, we used the forward primer 18S-F2 (5′-GGAAGGGTTGTATTTATTAGATAAAG-3′) and the reverse primer 18S-R2 (5′-AAGGAGTAAGGAACAACCTCCA-3′). The amplification conditions included initial denaturation at 94 °C for 3 min, followed by 35 cycles of denaturation at 94 °C for 30 s, annealing at 58 °C for 1 min and 30 s, extension at 72 °C for 2 min, and final extension at 72 °C for 7 min, resulting in a secondary product of 840 bp [28].

The subtyping of *Cryptosporidium* was performed using the nested PCR amplification of the gp60 gene. In the first PCR step, primers GP60-F1 (5′-ATAGTCTCCGCTGTATTC-3′) and GP60-R1 (5′-TCCGCTGTATTCTCAGCC-3′) were used. The amplification program included initial denaturation at 95 °C for 3 min, followed by 35 cycles of denaturation at 94 °C for 45 s, annealing at 50 °C for 45 s, extension at 72 °C for 60 s, and final extension at 72 °C for 10 min, resulting in a primary product of 1250 bp. In the second reaction, forward primer GP60-F2 (5′-GGAAGGAACGATGTATCT-3′) and reverse primer GP60-R2 (5′-GCAGAGGAACCAGCATC-3′) were used under the same conditions as in the first step. Amplification yielded a secondary product of 850 bp [29]. 

The amplified products were visualized on a 1% agarose gel and observed under UV light. All positive PCR products were sent to the commercial laboratory Microsynth Seqlab (Vienna, Austria) or SEQme (Dobříš, Czech Republic) for purification and sequencing in both directions, using identical primers to those that were used in the second steps of both PCR reactions for the 18S rRNA and gp60 genes. The sequencing was performed by the Sanger sequencing method. The resulting sequences were analyzed and edited using the software MEGA X version 10.1.5 build 10191107 [30]. The assembly of the nucleotide sequences was carried out using the Gene Tool Lite 1.0 software (BioTools Inc., Jupiter, FL, USA). The consensus sequences were compared with the sequences deposited in GenBank by applying the nucleotide BLAST algorithm at the National Center for Biotechnology Information (NCBI). The sequences from this study have been deposited in GenBank under unique accession numbers for the 18S rRNA gene (PP897358-PP897365) and the gp60 genes (PP916252-PP91627) for *Cryptosporidium* spp. 

The molecular identification of *Cryptosporidium* spp. was further confirmed by phylogenetic analysis using the maximum likelihood method with a minimum of 1000 bootstrap replications. For phylogenetic analyses, the nucleotide sequences obtained in this study and other *Cryptosporidium* spp. sequences from GenBank were used in the software MEGA X [30].

## 3. Results

### 3.1. Ziehl–Neelsen Staining Method

**Figure 1:**
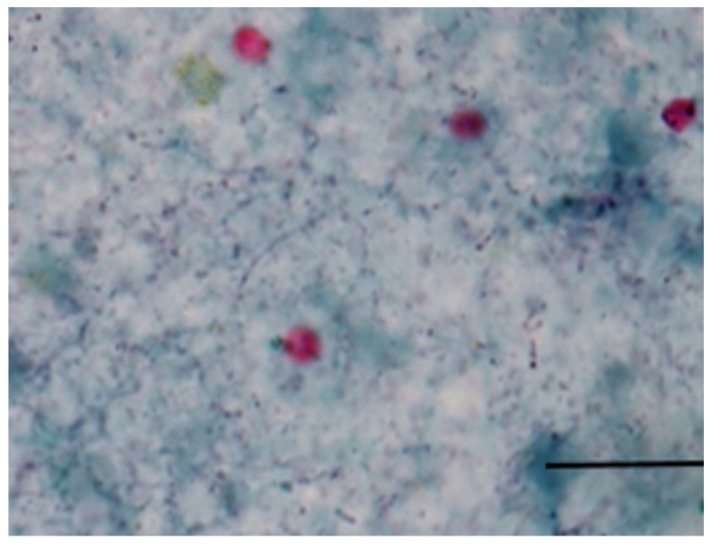
Oocysts of *Cryptosporidium* spp. according to Ziehl–Neelsen staining method; bar—20 µm.

In the first week, the microscopic observation of fecal smears did not confirm the presence of *Cryptosporidium* spp. oocysts. In the second week, *Cryptosporidium* spp. was confirmed microscopically in 15 samples (62.5%) out of a total of 24. In the third week, it was detected in two samples; in the fifth week, it was found in one sample. Sampling was not conducted in the fourth week (Figure 1) (Table 1 and Table 2).

### 3.2. ELISA Test 

In the first week, the presence of the *Cryptosporidium* spp. coproantigen was detected in two calves. Throughout the study period, the highest incidence of cryptosporidiosis was observed in the second week of life, while the antigen was confirmed in 22 (91.6%) calves. In the third and fifth weeks, the coproantigen was detected in only three calves; interestingly, these were different calves (Table 1 and Table 2).

Using the flotation method, the fecal samples were repeatedly examined for the presence of *Giardia duodenalis* cysts during all weeks. In the third week of the calves’ lives, positive findings of *Giardia duodenalis* cysts were observed. Additionally, in the fifth week, in addition to *Giardia duodenalis*, another protozoan parasite, *Eimeria* spp., was diagnosed (Table 3).

### 3.3. Molecular Identification and Phylogenetic Analysis of Cryptosporidium spp. and Subtypes

**Figure 2:**
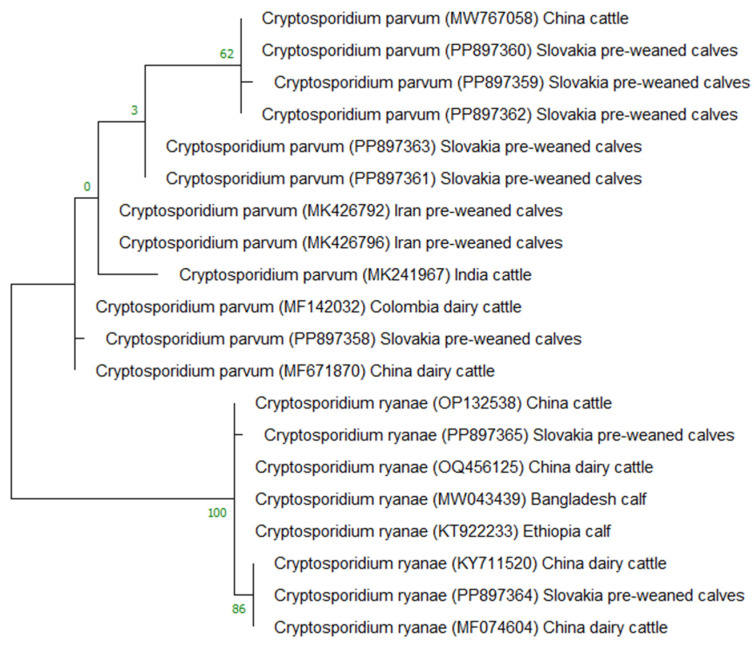
Phylogenetic tree constructed using the maximum likelihood method and Tamura–Nei model and depicting the relationships among *C. parvum* and *C. ryanae* based on the small subunit 18S rRNA gene sequence data available in the GenBank database.

The molecular analysis and sequencing of a fragment of the small subunit 18S rRNA gene (ssu rRNA) identified two species of *Cryptosporidium*, namely *C. parvum* (PP897358-PP897363) and *C. ryanae* (PP897364 and PP897365), in isolates taken from pre-weaning calves in various weeks post-birth. In all genomic sequences in the present study, the sequences for the 18S rRNA gene, deposited under numbers PP897358-PP897363, were compared in GenBank to the *C. parvum* MK426796, MK426792, MK241967, MW767058, MF671870, and MF142032 sequences, with the identity ranging from 99.80% to 100%. The sequence numbers PP897364 and PP897365 for 18S rRNA were compared in GenBank to the *C. ryanae* OQ456125, OP132538, MW043439, KT922233, KY711520, and MF074604, with the identity ranging from 99.88% to 100%. For the subgenotyping analysis, the sequence typing of the 60 kDa glycoprotein (gp60) gene was used. In the analysis of the gp60 gene, all sequences in this study (PP916252-PP916276) exhibited 99.64–100% nucleotide identity among sequences KC995129, MH796385, AM988863, and EF073050 of *C. parvum* and the subtype IIaA17G1R1 deposited in GenBank. In two cases, in the second week post-birth, the study detected a co-infection with *C. parvum* and *C. ryanae.* The phylogenetic analysis of the small subunit 18S rRNA gene, constructed using the maximum likelihood method and the Tamura–Nei model, organized the sequences in the present study (PP897358-PP897365) as well as the sequences available in GenBank (MK426796, MK426792, MK241967, MW767058, MF671870, MF142032, OQ456125, OP132538, MW043439, KT922233, KY711520, MF074604) into two distinct clusters for *C. parvum* and *C. ryanae* (Figure 2). 

## 4. Discussion

Ruminants are common hosts of *Cryptosporidium* spp., and contact with them represents a major risk factor for cryptosporidiosis infection in other animals or humans [3]. In this study, the dynamics of *Cryptosporidium* species was monitored and it was confirmed that the highest detection rate of infection occurred in the second week of the calves’ lives. This study found that the dominant species in calves up to five weeks old was the zoonotic *C. parvum*, which has also been confirmed by studies from various countries with intensive animal husbandry, in which these species were dominant in pre-weaned calves [31,32,33,34,35,36]. PCR amplification targeting the 18S rRNA gene also demonstrated the presence of *C. ryanae* on a farm in the eastern part of Slovakia in calves in their second week of life. Studies have indicated that *C. ryanae* is the dominant species primarily in post-weaned calves and young cattle; however, in certain regions of Sweden, China, and Sudan, *C. ryanae*, along with *C. bovis*, has been isolated from pre-weaned diarrheic and healthy calves [37,38,39,40,41,42]. This study confirms the first occurrence of *C. ryanae* in newborn calves in Slovakia.

Since the identification of *C. parvum* at the subtype level is essential from an epidemiological perspective with regard to cryptosporidiosis, all positive isolates at the gp60 locus were characterized and the presence of subtype IIaA17G1R1 was confirmed in all samples. This subtype had previously been identified in calves in Slovakia [43,44]. Additionally, subtype IIaA17G1R1 was diagnosed in Slovakia in hemato-oncological patients, further confirming its zoonotic potential [45]. The first report of the occurrence of the IIaA10G1R1, IIaA11G2R1, IIaA12G2R1, IIaA13G1R1, and IIaA14G1R1 animal subtypes of *C. parvum* in humans in Slovakia was confirmed by Hatalová et al., 2019 [46]. The occurrence of the IIaA17G1R1 subtype has been reported in various European countries, including Hungary, Sweden, the United Kingdom, Poland, and Germany [32,37,47,48,49]. The IIaA17G1R1 subtype, along with the IIaA16G1R1 subtype, has not only been identified in calves but also confirmed in sheep, pigs, lambs, dogs, horses, and donkeys [50,51,52,53,54,55]. The fact that these zoonotic subtypes have been isolated from various hosts increases the risk of *C. parvum* transmission from cattle to other animals and humans.

In the neighboring Czech Republic, according to the study by Ondráčková et al., 2009, the highest prevalence of *Cryptosporidium* spp. in cattle was found in those aged 12 to 18 months. A total of 995 samples were examined, with the predominant species being *C. andersoni* (4.1%), *C. bovis* (0.2%), and *C. parvum* (0.1%). The subtyping of the *C. parvum* species revealed the IIaA16G1R1 subtype [56]. Another study examining the prevalence of species in calves up to 2 months old confirmed the same species’ occurrence: *C. parvum* in 137 samples, *C. andersoni* in 21 samples, and *C. bovis* in 3 samples out of a total of 750 samples. The species *C. ryanae* was not confirmed in the calves. The subtyping of *C. parvum* confirmed the subtypes IIaA15G2R1, A16G1R1, A22G1R1, A18G1R1, and A15G1R1 [57]. An Austrian study in calves up to 180 days old confirmed the species *C. parvum* in 69 calves, *C. ryanae* in 11, and *C. bovis* in 7 out of a total of 177. The sequencing of the gp60 locus for *C. parvum* confirmed the subtypes IIaA15G2R1, IIaA21G2R1, IIaA19G2R1, and IIaA14G1R1 [58]. A Polish study reports the prevalence of cryptosporidiosis in cattle of 45.3% out of a total of 1601 tested animals, sampled from calves up to 4 months old, between the years 2014 and 2018. The dominant species were *C. bovis* and *C. ryanae*, with *C. parvum* being the third most common species [59].

The percentage of positive samples that were confirmed by commercially available ELISA tests, compared to the modified Ziehl–Neelsen staining method, corresponds to findings from other studies that have indicated that ELISA methods are more sensitive than microscopic methods [60,61]. Felefel et al., 2023 reported the lower sensitivity of the modified Ziehl–Neelsen method (19.23%) compared to coproantigen confirmation by the ELISA test (32.5%). Conversely, the study by Khurana et al., 2012 indicated that Auramine ‘O’-phenol staining (fluorescent staining, AP) is a highly sensitive method compared to the ELISA method [62]. 

Since calves can shed extremely large amounts of resistant and immediately infectious cysts into the surrounding environment, there is a high probability of infecting other calves. Delafosse et al., 2015 identified the risk factors affecting infection in calves. Multivariate analysis confirmed that the walls of the buckets were highly contaminated with oocysts, which were difficult to remove by applying standard washing procedures. This led to the subsequent contamination of the food placed in the buckets. Similarly, the early separation of calves from their mothers, due to which calves suckle each other (cross-suckling) or suckle other objects in the housing area, may cause possible oocyst infection [63]. The prevention of cryptosporidiosis is influenced by several factors, including the age of the calves, their overall health status, the colostrum intake, the feed quality, the water sources, the housing conditions, and the presence of other diarrheal diseases [64,65]. 

The control of cryptosporidiosis on farms can only be managed by combining good hygiene management with efficient preventive medications. Although the licensed drug that has been approved for the treatment of cryptosporidiosis in calves is halofuginone lactate, paromomycin has also proven to be efficient in the treatment of acute infections in cattle. For newborn calves, treatment with halofuginone lactate is recommended orally during the first seven days of life at a dose of 100 µg per kg of body weight [66,67,68]. 

In the present study, the treatment of cryptosporidiosis was not initiated due to the good clinical condition of the calves. After the second week, in which the incidence of the disease was the highest, the disease gradually self-resolved, as indicated by the declining infection rate observed in the following weeks. According to the farm’s veterinarian, hygiene measures were implemented to reduce the risk of infection spread. Thomson et al., 2017 noted that, in newborn calves, the disease may self-resolve due to the sufficient absorption of colostral antibodies, while maintaining the animals in a warm and dry environment, with supportive treatments and without other possible co-infections with other gastrointestinal pathogens [69]. 

Calves are one of the main reservoirs of zoonotic *Cryptosporidium* spp. species and contact with them can pose a possible risk of infection, especially for farm workers, animal technicians, veterinarians, and veterinary medicine students. Immunodeficient individuals, children, the elderly, and AIDS patients are particularly at risk [70,71]. 

The disease is of great importance from a public health perspective, highlighting the need for broader research and the identification of infection sources of *Cryptosporidium* spp. species and their genotypes.

## Figures and Tables

**Table 1:** Detection of *Cryptosporidium* spp. by Ziehl–Neelsen staining method and ELISA test.

No.	1st Week		2st Week		3st Week		5st Week	
	Ziehl–Neelsen	Elisa	Ziehl–Neelsen	Elisa	Ziehl–Neelsen	Elisa	Ziehl–Neelsen	Elisa
1.	-	-	+	+	-	-	-	-
2.	-	-	-	+	-	-	-	-
3.	-	-	-	+	-	-	-	-
4.	-	-	+	+	+	+	-	-
5.	-	-	+	+	-	-	-	-
6.	-	+	+	+	-	-	-	-
7.	-	+	-	-	-	-	-	-
8.	-	0	0	0	0	0	0	0
9.	-	-	+	+	-	-	-	+
10.	-	-	+	-	-	-	-	+
11.	-	-	-	+	-	-	-	-
12.	-	-	-	+	-	-	-	-
13.	-	-	+	+	+	+	+	+
14.	-	-	+	+	-	-	-	-
15.	-	-	+	+	-	-	-	-
16.	-	-	-	+	-	-	-	-
17.	-	-	+	+	-	-	-	-
18.	-	-	+	+	-	-	-	-
19.	-	-	-	+	-	-	-	-
20.	-	-	-	+	-	-	-	-
21.	-	-	+	+	-	-	-	-
22.	-	-	-	+	-	-	-	-
23.	-	-	+	+	-	+	-	-
24.	-	-	+	+	-	-	-	-
25.	-	-	-	+	-	-	-	-

- (negative); + (positive); 0 (no sample).

**Table 2:** Comparison of Ziehl–Neelsen staining method and ELISA test in each week.

	1st Week (0–7 Days)	2nd Week (7–14 Days)	3rd Week (14–21 Days)	4th Week (21–28 Days)	5th Week (28–35 Days)
Ziehl–Neelsen stain method	0%	62.5%	4.1%	-	4.1%
ELISA test	8%	91.6%	12.5%	-	12.5%

**Table 3:** Incidence of Coinfections of *Cryptosporidium* spp. detected by ELISA method and other parasites by flotation method.

No.	1st Week	2st Week	3st Week	4st Week	5st Week
1.	-	*Cryptosporidium* spp.	*Giardia duodenalis*	no sampling	-
2.	-	*Cryptosporidium* spp.	-	no sampling	-
3.	-	*Cryptosporidium* spp.	-	no sampling	*Giardia duodenalis*
4.	-	*Cryptosporidium* spp.	*Cryptosporidium* spp.	no sampling	*Eimeria* spp.
5.	-	*Cryptosporidium* spp.	*Giardia duodenalis*	no sampling	*Giardia duodenalis*
6.	*Cryptosporidium* spp.	*Cryptosporidium* spp.	-	no sampling	*Eimeria* spp.
7.	*Cryptosporidium* spp.	-	-	no sampling	*Eimeria* spp.
8.	-	no sampling	no sampling	no sampling	no sampling
9.	-	*Cryptosporidium* spp.	*Giardia duodenalis*	no sampling	*Cryptosporidium* spp.
10.	-	-	*Giardia duodenalis*	no sampling	*Cryptosporidium* spp., *Giardia duodenalis*
11.	-	*Cryptosporidium* spp.	*Giardia duodenalis*	no sampling	*Eimeria* spp.
12.	-	*Cryptosporidium* spp.		no sampling	-
13.	-	*Cryptosporidium* spp.	*Cryptosporidium* spp., *Giardia duodenalis*	no sampling	*Cryptosporidium* spp.
14.	-	*Cryptosporidium* spp.	-	no sampling	-
15.	-	*Cryptosporidium* spp.	-	no sampling	-
16.	-	*Cryptosporidium* spp.	-	no sampling	*Giardia duodenalis*
17.	-	*Cryptosporidium* spp.	-	no sampling	*Eimeria* spp.
18.	-	*Cryptosporidium* spp.	-	no sampling	-
19.	-	*Cryptosporidium* spp.	-	no sampling	-
20.	-	*Cryptosporidium* spp.	-	no sampling	-
21.	-	*Cryptosporidium* spp.	-	no sampling	*Giardia duodenalis*
22.	-	*Cryptosporidium* spp.	*Giardia duodenalis*	no sampling	-
23.	-	*Cryptosporidium* spp.	*Cryptosporidium* spp.	no sampling	*Giardia duodenalis, Eimeria* spp.
24.	-	*Cryptosporidium* spp.	*Giardia duodenalis*	no sampling	*Eimeria* spp.
25.	-	*Cryptosporidium* spp.	*Giardia duodenalis*	no sampling	*Eimeria* spp.

## Data Availability

The original contributions presented in the study are included in the article, further inquiries can be directed to the corresponding author.
